# Environmental Stability of Enveloped Viruses Is Impacted by Initial Volume and Evaporation Kinetics of Droplets

**DOI:** 10.1128/mbio.03452-22

**Published:** 2023-04-10

**Authors:** Andrea J. French, Alexandra K. Longest, Jin Pan, Peter J. Vikesland, Nisha K. Duggal, Linsey C. Marr, Seema S. Lakdawala

**Affiliations:** a Department of Microbiology & Molecular Genetics, University of Pittsburgh School of Medicine, Pittsburgh, Pennsylvania, USA; b Department of Civil and Environmental Engineering, Virginia Tech, Blacksburg, Virginia, USA; c Department of Biomedical Sciences and Pathobiology, Virginia-Maryland College of Veterinary Medicine, Virginia Tech, Blacksburg, Virginia, USA; d Center for Vaccine Research, University of Pittsburgh School of Medicine, Pittsburgh, Pennsylvania, USA; e Department of Microbiology and Immunology, Emory University, Atlanta, Georgia, USA; Icahn School of Medicine at Mount Sinai

**Keywords:** influenza, SARS-CoV-2, survival, stability, persistence, droplets, volume, size, droplet volume, influenza virus, virus decay, virus stability

## Abstract

Efficient spread of respiratory viruses requires the virus to maintain infectivity in the environment. Environmental stability of viruses can be influenced by many factors, including temperature and humidity. Our study measured the impact of initial droplet volume (50, 5, and 1 μL) and relative humidity (RH; 40%, 65%, and 85%) on the stability of influenza A virus, bacteriophage Phi6 (a common surrogate for enveloped viruses), and severe acute respiratory syndrome coronavirus 2 (SARS-CoV-2) under a limited set of conditions. Our data suggest that the drying time required for the droplets to reach quasi-equilibrium (i.e., a plateau in mass) varied with RH and initial droplet volume. The macroscale physical characteristics of the droplets at quasi-equilibrium varied with RH but not with the initial droplet volume. Virus decay rates differed between the wet phase, while the droplets were still evaporating, and the dry phase. For Phi6, decay was faster in the wet phase than in the dry phase under most conditions. For H1N1pdm09, decay rates between the two phases were distinct and initial droplet volume had an effect on virus viability within 2 h. Importantly, we observed differences in virus decay characteristics by droplet size and virus. In general, influenza virus and SARS-CoV-2 decayed similarly, whereas Phi6 decayed more rapidly under certain conditions. Overall, this study suggests that virus decay in media is related to the extent of droplet evaporation, which is controlled by RH. Importantly, accurate assessment of transmission risk requires the use of physiologically relevant droplet volumes and careful consideration of the use of surrogates.

## INTRODUCTION

Respiratory viruses, such as influenza A virus and severe acute respiratory syndrome coronavirus 2 (SARS-CoV-2), contribute to high morbidity and mortality. These viruses must remain infectious in the environment for transmission to the next host to succeed. Understanding how environmental, host, and virus factors impact the stability of expelled virus will lead to a better assessment of virus transmission risk and ways to reduce it.

Many factors can impact virus stability in the environment, including virion structure, temperature, relative humidity (RH), droplet composition, solute concentration, and fomite surface material (1–5). However, the relationship between droplet volume and virus stability is not well understood, even though droplet volume plays an important role in virus transmission. Droplet volume impacts the distance traveled by respiratory expulsions (6). Smaller droplets, or aerosols, can travel further from the infected host, while larger droplets settle to the ground more quickly due to their increased mass (6). Droplet volume can also be a determinant of host infection site, as particles smaller than 10 μm in diameter are more likely to deposit deeper in the respiratory tract (7). Given the importance of droplet volume to initiating infection, understanding how volume affects virus stability is critical to mitigating transmission of respiratory viruses such as influenza virus and coronaviruses.

Studies measuring virus stability in the environment typically use one of two methods to generate droplets: nebulizers to produce aerosols or pipettes to create droplets with as much as 50 μL per droplet. While large droplets are commonly used to assess environmental virus stability, they do not mimic the physiological volume of a droplet created by an expulsion. The vast majority of expelled droplets from the respiratory tract are less than 0.5 μL in volume (approximately 1 mm in diameter for a sphere); a droplet of 50 μL (approximately 4.6 mm in diameter for a sphere) is about 5 times larger and 100 times greater in volume (8). Studies measuring the stability of SARS-CoV-2 on surfaces have examined the virus in 5-μL (9), 10-μL (10), 20-μL (11), or 50-μL (2, 3, 12) droplets, all larger than most expelled droplets. These initial studies of SARS-CoV-2 stability were used widely for policy decisions and to assert the importance of contaminated surfaces to transmission. However, little work has been done to understand whether virus decay in large droplets is representative of decay in smaller, more physiologically relevant droplet volumes.

This study primarily used the 2009 pandemic influenza H1N1 virus (H1N1pdm09, A/CA/07/2009) and bacteriophage Phi6, a commonly used virus surrogate, to examine environmental stability of enveloped viruses in three different droplet volumes at three different RHs over time. Specifically, we measured the viability of each virus in 50-, 5-, and 1-μL droplets on surfaces over time at 40%, 65%, and 85% RH. We observed that virus within smaller droplets decays quickly regardless of RH, while virus decay occurs more slowly in larger droplets. We also explored droplet evaporation rates and found that virus decay is closely correlated with the extent of evaporation, which is likely a proxy for the solute concentration in the droplet. Additionally, limited experiments with SARS-CoV-2 showed that influenza virus decayed similarly to SARS-CoV-2 at an intermediate (55 to 60%) RH in 50-, 5-, and 1-μL droplets. Overall, our results suggest that virus stability studies should use smaller, more physiologically relevant droplet volumes and should recognize the limitations of surrogate viruses.

## RESULTS

### Relative humidity alters morphology of evaporating droplets and drying kinetics.

**FIG 1:**
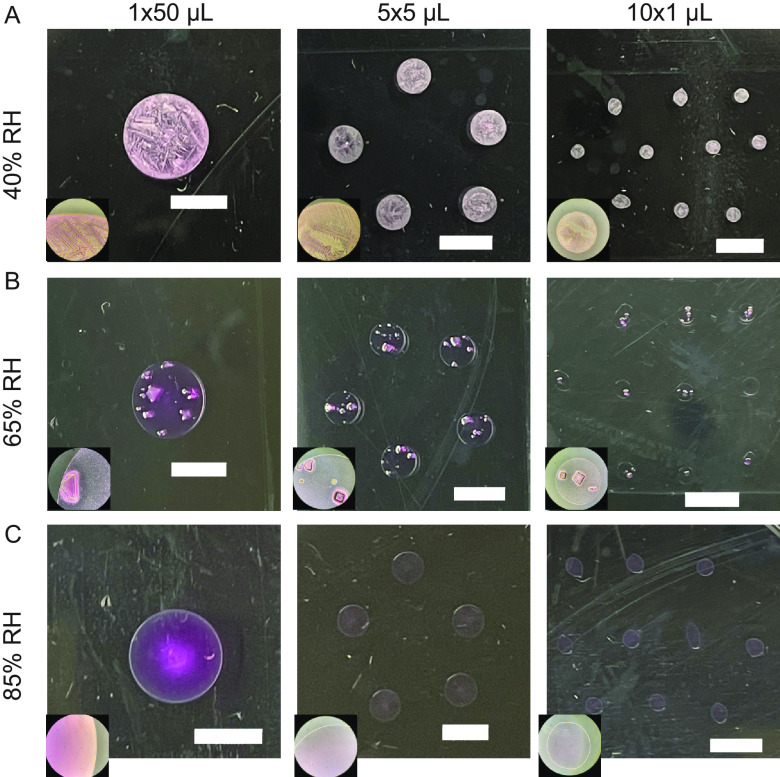
Macroscale physicochemical characteristics of DMEM droplets vary with RH but not initial volume. Inset images taken with 10× objective. Scale bars indicate 5 mm. (A) At 40% RH, droplets become concentrated at the border and develop interior feather-like crystals. (B) At 65% RH, droplets develop distinct crystals within the interior. (C) At 85% RH, droplets maintain moisture and do not crystallize.

We expect the physical and chemical characteristics of droplets to influence decay of viruses within each droplet. Some of these physical and chemical characteristics may be reflected in the morphology of droplets after they have dried (5). Furthermore, fluid dynamics within droplets could lead to increased aggregation of virus, which can enhance virus stability (13, 14). We investigated whether droplet morphology and drying pattern at 24 h differed between 1 × 50-, 5 × 5-, or 10 × 1-μL droplets (i.e., 1 droplet with a volume of 50 μL, 5 droplets with a volume of 5 μL, or 10 droplets with a volume of 1 μL). Droplets of medium (Dulbecco’s modified Eagle medium [DMEM]) were placed on polystyrene plastic and incubated at 40%, 65%, or 85% RH for 24 h (Fig. 1; see also Movies S1 to S3 at https://doi.org/10.6084/m9.figshare.21711119, https://doi.org/10.6084/m9.figshare.21711122, and https://doi.org/10.6084/m9.figshare.21711116). These RHs were selected to match values in other published work (3). We report RH instead of absolute humidity because the former is more directly related to virus inactivation (15). Droplet morphology in droplets containing virus was the same as in droplets of media alone (data not shown). The effect of RH on dried droplet morphology was independent of initial droplet volume (Fig. 1). This suggests that observed differences in viral decay by droplet size would not be due to final physicochemical differences. Our results show that the droplet drying pattern at 24 h depends on RH but not initial droplet volume.

Evaporation leads to the concentration of solutes, which can influence virus stability in droplets (15, 16). To investigate the drying kinetics, we recorded the mass of 1 × 50-μL, 5 × 5-μL, or 10 × 1-μL droplets of DMEM containing Phi6 for 24 h at ambient temperature and three RHs: 40%, 65%, and 85% RH (as shown in Fig. 1). The droplets at all RHs lost mass linearly over time before reaching a plateau, referred to as a quasi-equilibrium (see Fig. S1 and Tables S1 and S2 in the supplemental material) (2). We defined this state as quasi-equilibrium because it is likely that very slow evaporation continues over a much longer time scale until complete dryness occurs or until a crust or shell forms that blocks further water loss. To simplify this discussion, we refer to the time period before this as the wet phase and the period after this as the dry phase. The drying time required for the droplets to reach quasi-equilibrium ranged from 0.5 h for 1-μL droplets at 40% RH to 11 h for 50-μL droplets at 85% RH. These data indicate that droplets of different volumes undergo different drying kinetics. If the kinetics of drying affect virus stability, then it could differ by initial droplet volume.

10.1128/mbio.03452-22.1 FIG S1 Initial droplet volume impacts drying kinetics. (A to C) Mass normalized to starting mass for all droplet volumes at 40% (A), 65% (B), or 85% (C) RH over time. (D) Summary data showing the time (mean and standard deviation; *n* = 2) for droplets to reach quasi-equilibrium at each RH. Droplet mass was measured on a microbalance in an environmental chamber and recorded every minute. Download FIG S1, PDF file, 0.1 MB. Copyright © 2023 French et al. 2023 French et al. https://creativecommons.org/licenses/by/4.0/ This content is distributed under the terms of the Creative Commons Attribution 4.0 International license.

10.1128/mbio.03452-22.4 TABLE S1 The evaporation rates for 1 × 50-μL, 5 × 5-μL, or 10 × 1-μL droplets at 40%, 65%, or 85% RH were determined by fitting a line to the mass over time. Download Table S1, PDF file, 0.1 MB. Copyright © 2023 French et al. 2023 French et al. https://creativecommons.org/licenses/by/4.0/ This content is distributed under the terms of the Creative Commons Attribution 4.0 International license.

10.1128/mbio.03452-22.5 TABLE S2 Droplets (1 × 50 μL, 5 × 5 μL, or 10 × 1 μL) were weighed over time. The time of quasi-equilibrium was determined and the mass at quasi-equilibrium was measured. Download Table S2, PDF file, 0.04 MB. Copyright © 2023 French et al. 2023 French et al. https://creativecommons.org/licenses/by/4.0/ This content is distributed under the terms of the Creative Commons Attribution 4.0 International license.

### Virus decay is more sensitive to relative humidity in large droplets.

**FIG 2:**
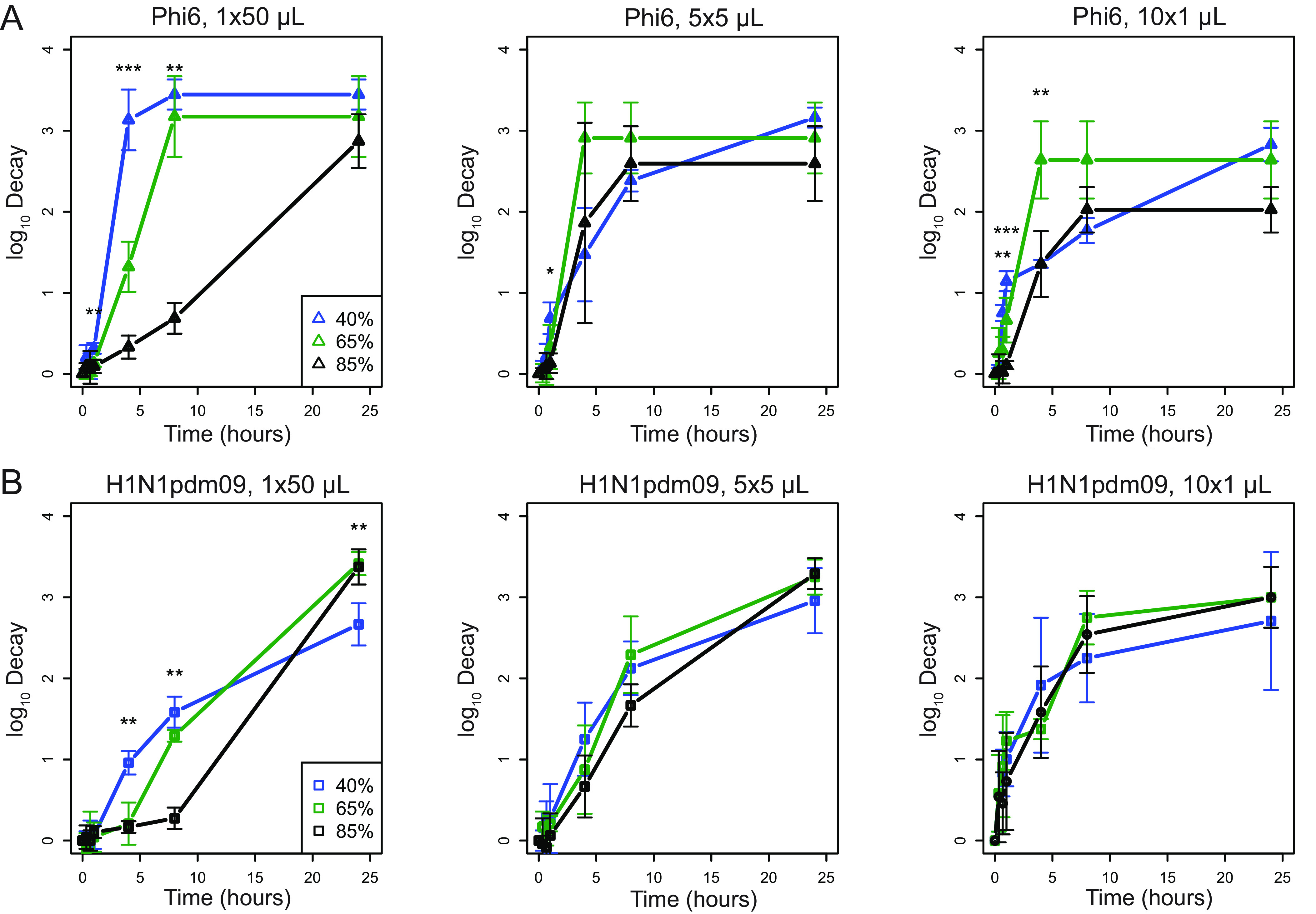
Virus decay varies more with relative humidity in large droplets than in small droplets. (A) Titers of Phi6 in 1 × 50-μL, 5 × 5-μL, or 10 × 1-μL droplets compared at 40%, 65%, and 85% RH in terms of log_10_ decay. (B) Titers of H1N1pdm09 in 1 × 50-μL, 5 × 5-μL, or 10 × 1-μL droplets compared at 40%, 65%, and 85% RH in terms of log_10_ decay. Error bars show standard deviations. Asterisks indicate significant differences between two or three RHs. For all graphs, *n* = 3 except at 1 h, where H1N1pdm09 *n* = 6. One-way ANOVA were conducted between the RHs at each time point. A Tukey honestly significant difference (HSD) test was conducted to determine between which RHs the significant differences (*P* < 0.05) occurred. Statistical details can be found in Table S3.

To directly examine how RH and droplet volume impact virus stability in the environment, we applied virus in droplets of different volumes to a polystyrene surface and quantified recovery of infectious virus over time (17). We compared decay of H1N1pdm09 and Phi6 at each RH in 1 × 50-μL, 5 × 5-μL, or 10 × 1-μL droplets at 40%, 65%, and 85% RH (Fig. 2 and Fig. S2). In 50-μL droplets, Phi6 decayed fastest at 40% RH and slowest at 85% RH (Fig. 2A). The impact of RH on the decay of H1N1pdm09 in 50-μL droplets over the first 8 h was similar to that for Phi6 but less pronounced, with the greatest decay occurring at 40% RH (Fig. 2B). Decay of H1N1pdm09 in the 1 × 50-μL droplet was first detected at 4 h at 40% RH, 8 h at 65% RH, and 24 h at 85% RH, indicating that early virus decay was inversely related to RH (i.e., more decay at lower RH) in large (50-μL) droplets (Table S4). Decay of Phi6 in 5-μL droplets differed by RH only at 1 h, when decay at 40% was greater than at 85%. In 1-μL droplets, decay differed between 40 min and 4 h by RH but was not significantly different at 8 h or afterward (Fig. 2A). H1N1pdm09 in 1-μL and 5-μL droplets decayed at similar rates within each droplet volume regardless of RH (Fig. 2B). Phi6 was more unstable after drying in the intermediate RHs, whereas H1N1pdm09 tended to be more stable. This accounts for differences in decay at the smaller droplet sizes. These findings show that the impact of RH on virus decay in droplets depends on the virus and the initial volume of the droplets.

10.1128/mbio.03452-22.2 FIG S2 Environmental conditions of H1N1pdm09 droplets and Phi6 droplets were within 5% of targeted RH and maintained temperatures between 20 and 28°C. The RH and temperature of the environmental chamber were recorded every 15 min during H1N1pdm09 stability experiments at (A) 40% (A), 65% (B), and 85% (C) RH. The RH and temperature of the environmental chamber were recorded every minute during Phi6 stability experiments at 40% (D), 65% (E), and 85% (F) RH. The RH and temperature data for comparing decay of H1N1pdm09 at 60% (G) with SARS-CoV-2 at 55% (H) were recorded every 15 min or 1 min, respectively. Light green shows the temperature at each replicate, and dark green indicates the average temperature for the 3 independent replicates. Light blue shows the RH for each replicate, and dark blue indicates the average RH for the 3 independent replicates. (I) Absolute humidities were calculated for all experiments. The keys show the corresponding average RH for each condition instead of targeted RH. Conditions for data previously published by van Doremalen et al. (N Engl J Med 382:1564–1567, 2020, https://doi.org/10.1056/NEJMc2004973) are unknown. Download FIG S2, PDF file, 0.4 MB. Copyright © 2023 French et al. 2023 French et al. https://creativecommons.org/licenses/by/4.0/ This content is distributed under the terms of the Creative Commons Attribution 4.0 International license.

10.1128/mbio.03452-22.7 TABLE S4 Log_10_ decay for each virus was compared to 0 decay at each time point in 1 × 50-μL droplets. Download Table S4, PDF file, 0.05 MB. Copyright © 2023 French et al. 2023 French et al. https://creativecommons.org/licenses/by/4.0/ This content is distributed under the terms of the Creative Commons Attribution 4.0 International license.

10.1128/mbio.03452-22.6 TABLE S3 Log_10_ decay for each virus was compared between RH for each droplet volume, and *P* values were determined. Download Table S3, PDF file, 0.1 MB. Copyright © 2023 French et al. 2023 French et al. https://creativecommons.org/licenses/by/4.0/ This content is distributed under the terms of the Creative Commons Attribution 4.0 International license.

### Virus decay rates differ during the wet and dry phases and depend on droplet volume and virus.

**FIG 3:**
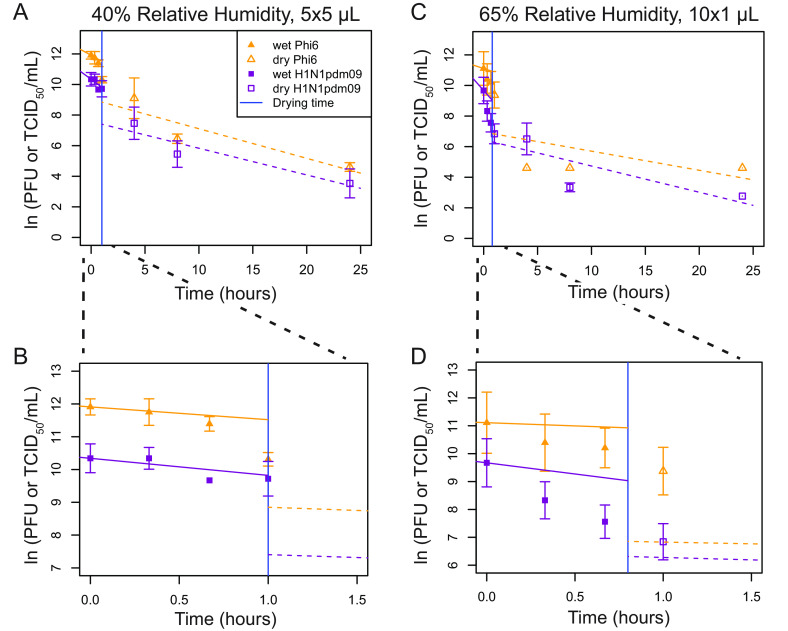
Mechanistic first-order decay modeling of viral decay in 5 × 5-μL droplets at 40% RH and 10 × 1-μL droplets at 65% RH shows that viral decay during the wet phase is greater than decay during the dry phase. (A to D) First-order exponential decay models, accounting for increasing solute concentrations over time during the wet phase, were fit to ln(PFU or TCID_50_/mL) over time for 5 × 5-μL droplets at 40% RH (A and B) or 10 × 1-μL droplets at 65% RH (C and D). In panels B and D, magnifications of panels A and C from 0 to 1.5 h are shown. For the wet phase, the fitted line represents the initial decay rate, i.e., what it would be if the droplets remained the same size and did not evaporate. Actual RHs were ±2% from targeted RHs (Fig. S2). For all graphs, *n* = 3, except at 1 h, where H1N1pdm09 *n* = 6. The vertical blue line indicates the time of transition from the wet phase to the dry phase. A *t* test was used to compare the slopes between the evaporation and dry phases for each virus at each droplet volume and between the phases for each virus at each droplet volume (*P* < 0.05). Statistical details can be found in Table S5.

**FIG 4:**
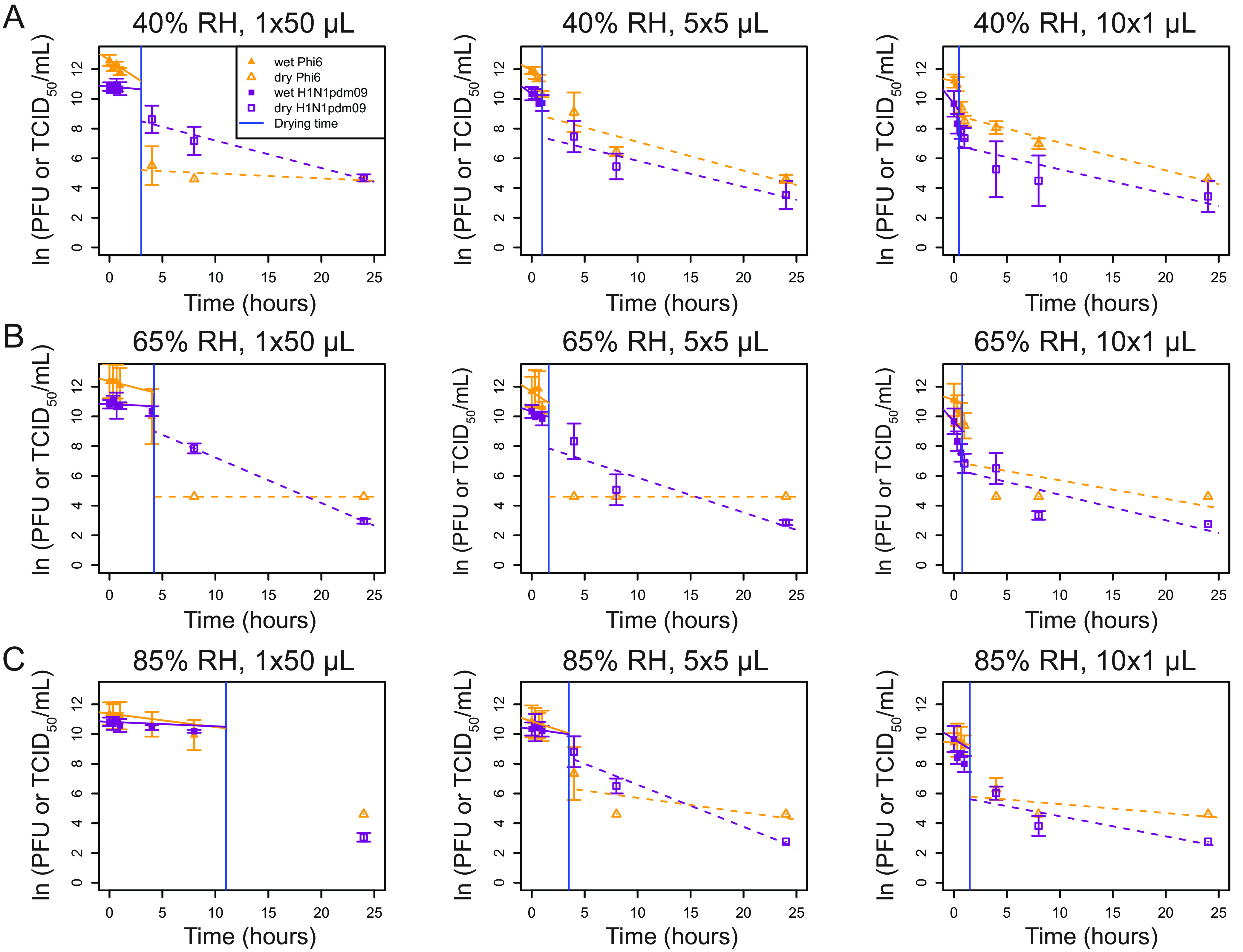
Mechanistic, first-order exponential decay modeling shows that the decay during the wet phase is greater or similar to the rate of decay the dry phase. (A to C) First-order exponential decay models, accounting for increasing solute concentrations over time during the wet phase, were fit to ln(PFU or TCID_50_/mL) over time for 1 × 50-μL, 5 × 5-μL, or 10 × 1-μL droplets at 40% RH (A), 65% RH (B), or 85% RH (C). For the wet phase, the fitted line represents the initial decay rate, i.e., what it would be if the droplets remained the same size and did not evaporate. Actual RH were ±2% from targeted RHs (Fig. S2). For all graphs, *n* = 3 except at 1 h, where H1N1pdm09 *n* = 6. The vertical blue line indicates the time of transition from the wet phase to the dry phase. A *t* test was used to compare the slopes between the evaporation and dry phases for each virus at each droplet volume and between the phases for each virus at each droplet volume (*P* < 0.05). Statistical details can be found in Table S5.

The pattern of decay for Phi6 and H1N1pdm09 appeared to be distinct for different droplet volumes. This led us to investigate whether drying time impacts virus decay and whether different viruses behave similarly across different droplet volumes. Virus decay often follows first-order kinetics (18). Following a previously developed mechanistic model of virus inactivation in droplets, we fit an exponential decay curve model (see Materials and Methods for details) to virus titers in droplets during the wet phase (prior to quasi-equilibrium) and a separate curve during the dry phase to create a biphasic model (Fig. 3 and 4 and Table 1) (4). The model accounts for changing solute concentrations in the droplets as they evaporate during the wet phase (2). Because of this, the model fit for the initial decay rate shown in the figures does not match the data points. Rather, the model indicates what the decay rate would be if the droplets remained the same size and did not evaporate throughout the wet phase.

**TABLE 1:** Exponential decay rate constants for Phi6 and H1N1pdm09

RH (%)	Initial size (μL)	Exponential decay rate constant^*a*^			
		Phi6		H1N1pdm09	
		Wet phase	Dry phase	Evaporation phase	Dry phase
40	50	0.47 ± 0.23	0.03 ± 0.04^+^	0.06 ± 0.13	0.19 ± 0.03^+^
40	5	0.39 ± 0.02*	0.19 ± 0.09*^+^	0.52 ± 0.16*	0.17 ± 0.06*^+^
40	1	0.14 ± NA	0.19 ± 0.03^+^	0.92 ± NA	0.16 ± 0.06^+^
65	50	0.18 ± 0.01^+^	<0.01 ± 0.00	0.03 ± 0.01^+^	0.31 ± 0.00
65	5	0.47 ± 0.23*	<0.01 ± 0.00*^+^	0.23 ± 0.15	0.23 ± 0.11^+^
65	1	0.23 ± 0.21	0.13 ± 0.14	0.80 ± 0.43	0.17 ± 0.08
85	50	0.09 ± 0.01^+^	NA ± NA	0.03 ± 0.01^+^	NA ± NA
85	5	0.23 ± 0.03	0.10 ± 0.11^+^	0.10 ± 0.17	0.28 ± 0.06^+^
85	1	0.08 ± 0.02	0.06 ± 0.07	0.45 ± 0.25	0.14 ± 0.08

a First-order exponential decay curves were fit to virus decay data of Phi6 and H1N1pdm09 in 1 × 50-μL, 5 × 5-μL, and 10 × 1-μL droplets at 40%, 65%, and 85% RH. The constant is determined by dividing 1 by the time in hours. Rates are shown plus/minus the standard errors. “NA” indicates that a line could not be fit due to only 1 point occurring after the quasi-equilibrium time. An asterisk indicates significant differences in slope between the wet and dry phases for the given size and virus. A superscript plus indicates a significant difference in slope between Phi6 and H1N1pdm09 for the given phase and size.

10.1128/mbio.03452-22.8 TABLE S5 Decay constants for each phase and virus were compared within each RH and droplet volume to characterize how phase and virus impact virus decay. Download Table S5, PDF file, 0.1 MB. Copyright © 2023 French et al. 2023 French et al. https://creativecommons.org/licenses/by/4.0/ This content is distributed under the terms of the Creative Commons Attribution 4.0 International license.

Virus decay appeared to be biphasic. In most cases for Phi6, decay was faster in the wet phase than in the dry phase. For H1N1pdm09, differences in decay rates between the two phases were not consistent. Figure 3 shows viability as a function of time for two conditions: 5 × 5-μL droplets at 40% RH (Fig. 3A and B) and 10 × 1-μL droplets at 65% RH (Fig. 3C and D). The insets show the detail during the first 1.5 h (Fig. 3B and D), when the droplets transitioned from wet to dry. Similar patterns were evident in most of the nine combinations of initial volume and RH for both viruses (Fig. 4 and Table 1).

Among the 18 total combinations of RH, initial droplet volume, and virus, there were 12 combinations for which the decay rate constant could be compared between the wet phase and dry phase. Decay rates were greater in magnitude during the wet phase than the dry phase in 7 of 12 cases and significantly greater in 3 of 12 of these cases (Table 1): Phi6 in 5 × 5-μL droplets at 40% RH (Fig. 3A), H1N1pdm09 in 5 × 5-μL droplets at 40% RH (Fig. 3A), and Phi6 in 5 × 5-μL droplets at 65% RH (Fig. 4B). Because there were only two time points during the wet phase for the 10 × 1-μL droplets at 40% RH and only one or two time points during the dry phase for 1 × 50-μL droplets at 65% and 85% RH, it was not possible to compare decay rates for these conditions (Table 1). Multivariate analysis, described in greater detail in the supplemental material, revealed a significant interaction between initial droplet volume and RH during wet-phase decay for H1N1pdm09 but not Phi6.

The decay rate constant was significantly higher for Phi6 than H1N1pdm09 in two cases during the wet phase and was significantly different in two cases—higher for H1N1pdm09 in both cases—during the dry phase (Fig. 4 and Table 1). Significant differences were not observed for the 1-μL droplets. These results indicate that different enveloped RNA viruses may decay differently.

### H1N1pdm09 decays similarly to SARS-CoV-2 at intermediate RH.

**FIG 5:**
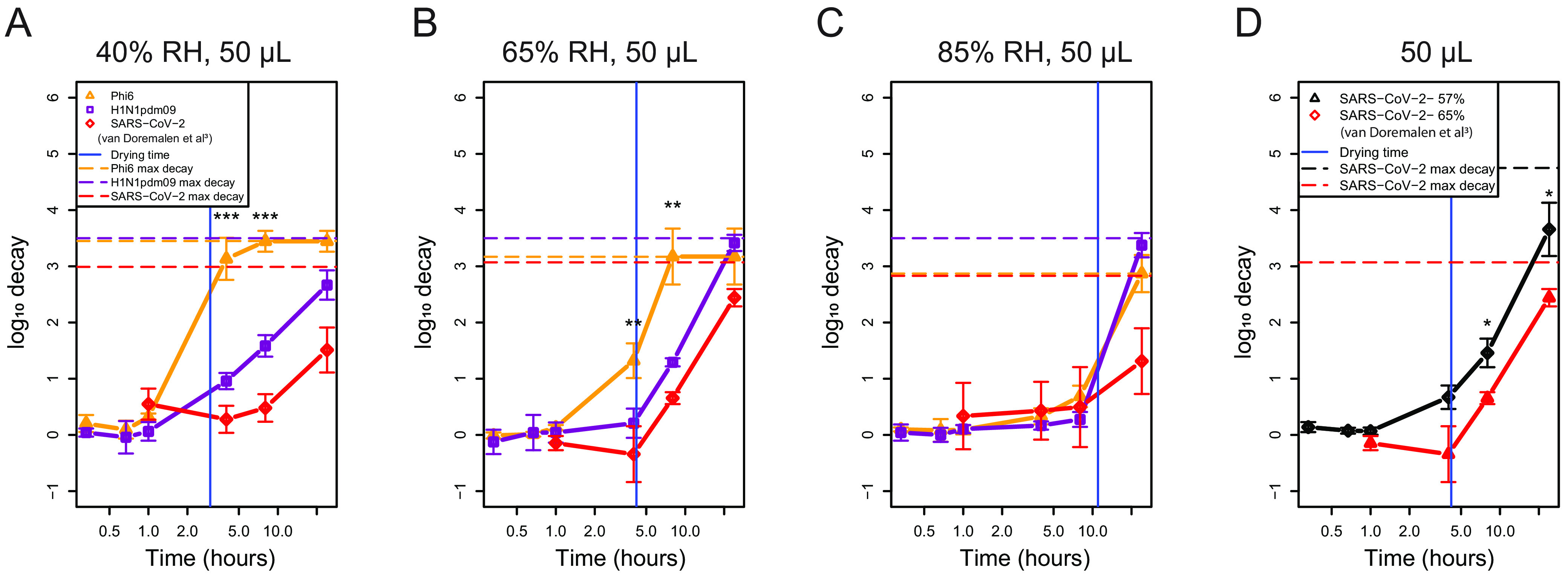
SARS-CoV-2 and H1N1pdm09 decay similarly across RH in 1 × 50-μL droplets. (A to C) SARS-CoV-2, H1N1pdm09, and Phi6 stability was measured at 40% (A), 65% (B), and 85% (C) RH in 50-μL droplets using SARS-CoV-2 data originally published by van Doremalen et al. (3). (D) Original data for SARS-CoV-2 at 55% were compared to the previously published data (3) at a targeted RH of 65%. Actual environmental conditions for original work can be found in Fig. S2. Environmental conditions for data of van Doremalen et al. for SARS-CoV-2 were not available. The vertical blue line indicates the time of transition from the wet phase to the dry phase. A one-way ANOVA and Tukey HSD test were used to determine statistical significance. Statistical details can be found in Table S6.

Given the observed differences in the decay rate constants of H1N1pdm09 and Phi6 (Fig. 4 and Table 1), we further investigated how the stability of these two enveloped RNA viruses compared to the stability of SARS-CoV-2 using both original and previously published data (3). To determine whether these viruses undergo similar patterns of decay at 40%, 65%, and 85% RH, we compared our results for H1N1pdm09 and Phi6 in 50-μL droplets to published results for SARS-CoV-2 (Fig. 5A to C and Table S6) (3). Consistent droplet composition, temperature, and RH between our study and the published results facilitated this comparison. While the published work started with 10^5^ 50% tissue culture infective doses (TCID_50_)/mL for SARS-CoV-2 and our viruses were 10^6^ PFU or TCID_50_/mL, we believe this difference to be negligible (1). There were significant differences for each pairwise comparison of the decay of H1N1pdm09, Phi6, and SARS-CoV-2 at 40% RH at 4 and 8 h; SARS-CoV-2 was most stable, followed by H1N1pdm09 and then Phi6 (Fig. 5A). At 65% RH, there were fewer differences: only Phi6 was significantly different (less stable) from H1N1pdm09 and SARS-CoV-2, again at 4 and 8 h (Fig. 5B). At 85% RH, there were no significant differences for the decay of any pairwise comparison (Fig. 5C). Significance at 24 h was not assessed due to virus decay reaching the limit of detection for at least one of the viruses tested. This suggests that in large (50-μL) droplets, virus-specific differences are greater at lower RH. To validate the use of the previously published data, we compared original SARS-CoV-2 experiments at a targeted 55% RH (actual 57% RH) to the previously published SARS-CoV-2 data at a targeted 65% RH (3) (actual RH not known) (Fig. 5D). Comparison of the original and published SARS-CoV-2 decay shows that similar trends and statistical differences are likely due to the difference in RH (Fig. 5D).

10.1128/mbio.03452-22.9 TABLE S6 Log_10_ decays within 1 × 50-μL droplets were compared between Phi6, H1N1pdm09, and SARS-CoV-2 at 40%, 65%, and 85% RH over time. Log_10_ decay within 50-μL, 5-μL, or 1-μL droplets were compared between H1N1pdm09 and SARS-CoV-2. Log_10_ decays of H1N1pdm09 and SARS-CoV-2 were compared across time for each droplet volume at 55 to 60% RH. Comparisons between H1N1pdm09 at 60% and 65% are also shown. Download Table S6, PDF file, 0.1 MB. Copyright © 2023 French et al. 2023 French et al. https://creativecommons.org/licenses/by/4.0/ This content is distributed under the terms of the Creative Commons Attribution 4.0 International license.

**FIG 6:**
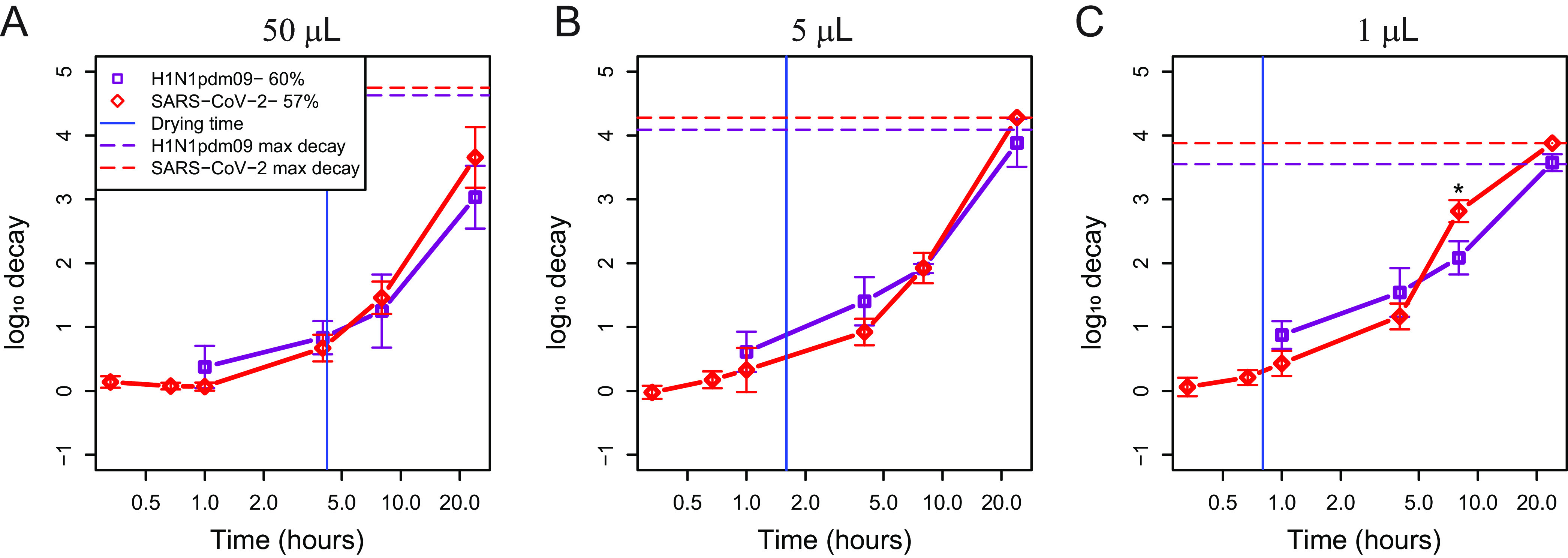
SARS-CoV-2 and H1N1pdm09 decay similarly across droplet volume at intermediate RH. (A to C) SARS-CoV-2 stability was compared to H1N1pdm09 stability at 55 to 60% RH in 1 or 2 × 50-μL (A), 5 × 5-μL (B), and 10 × 1-μL (C) droplets over time. The vertical blue line indicates the time of transition from the wet phase to the dry phase. The key indicates the average RH during experiments rather than the targeted RH. A one-way ANOVA was used to determine statistical significance. Statistical details can be found in Table S6. Experiments were conducted in 2 or 3 independent replicates each with technical duplicates. Data from both replicate studies are presented here.

To understand the role of droplet volume in decay of different enveloped respiratory viruses, we assessed titers of SARS-CoV-2 and H1N1pdm09 in 50-μL, 5 × 5-μL, and 10 × 1-μL droplets at 55% and 60% RH, respectively. Due to technical limitations, we were not able to test the exact same RH, but we consider these conditions to be similar, as their actual RHs were 57% and 60% (Fig. S2G to I). SARS-CoV-2 stability in 50-μL, 5-μL, and 1-μL droplets at 55% RH was similar to that of H1N1pdm09 at 60% RH (Fig. 6A to C and Table S6). While the decay of H1N1pdm09 in the 50-μL droplet at 65% RH appeared greater at 8 h than that of SARS-CoV-2, this difference was not significant (Fig. 5B). The log_10_ decay in 10 × 1-μL droplets between H1N1pdm09 and SARS-CoV-2 was significantly different only at 8 h, and their decays were similar again at 24 h. Taken together with Fig. 5, these results show that SARS-CoV-2 and H1N1pdm09 decay similarly at intermediate RH and that differences in virus decay may occur in larger droplets at low RH.

## DISCUSSION

The studies detailed here characterize the interplay of droplet volume and RH on the stability of three enveloped RNA viruses: Phi6, H1N1pdm09, and SARS-CoV-2. Our results showed that RH has a greater impact on viral decay in large (50-μL) droplets than in small (1-μL) droplets, that decay rates during the wet phase are greater than or similar to decay rates during the dry phase regardless of droplet size and RH, and that differences in virus decay are more common in 50-μL droplets than in 1-μL droplets and at low RH.

Our results raise questions about the relevance of prior studies on stability of viruses that employ large droplet volumes. For example, one study derived a half-life (~0.3-log decay) of 6.8 h for SARS-CoV-2 in 50-μL droplets on polypropylene plastic (3). Another used 5-μL droplets to evaluate the lifetime of SARS-CoV-2 on different materials and reported 0.5-log decay in 3 h and 1.1-log decay in 6 h on plastic, similar to the results shown here (Fig. 6B) (9). The conclusions reached within these studies might have differed had they used smaller, more physiologically relevant droplet volumes. In our study, after 4 h, we observed no significant decay in 50-μL droplets, ~1-log decay in 5-μL droplets, and ~1.5-log decay in 1-μL droplets at 55 to 60% RH. Over longer periods, the results converged, as we observed at least 3-log decay in all three droplet volumes after 24 h. These differences are likely controlled by physical and chemical properties of the droplets, as they undergo evaporation at different rates depending on their initial volume and ambient humidity.

We have attempted to study viruses in a more realistic droplet volume than those used in past research, but even a 1-μL droplet is at the extremely large end of the range of droplet volumes observed in respiratory emissions. During talking, coughing, and sneezing, droplets of this volume are emitted in much lower numbers, by many orders of magnitude, than those that are 100 to 1,000 times smaller in diameter and that behave as aerosols (6). While we observed differences between 1 × 50-μL and 10 × 1-μL droplets in this study, previous work has shown that virus in 10 × 1-μL droplets undergoes similar decay to that in aerosols at 23% to 98% RH and 22°C (19). Other techniques, such as a droplet-on-demand dispenser, are needed to study smaller droplet volumes on surfaces and determine whether there are fewer meaningful differences as droplets further decrease in size from 1 μL.

Our results also suggest caution in the use of surrogates to study the stability of pathogenic viruses and their potential for transmission. Surrogates that require fewer biosafety precautions are attractive for obvious reasons (20). They can be useful for evaluating sampling and analysis methods, studying physicochemical processes such as mechanisms of decay and transport in complex media, or eliciting trends in survival in complex media. For example, our group has used Phi6 to study the fate and transport of Ebola virus in wastewater systems and to examine how survival of viruses in droplets and aerosols varies with humidity and media composition (1, 19, 21, 22). However, we should be cautious about extrapolating survival times from surrogates to other viruses. In the present study, we found that Phi6 decayed more quickly than did influenza virus and SARS-Cov-2 under our experimental conditions. In order to place viruses in the same droplet solution, Phi6 had to be ultracentrifuged, which may have contributed to differences in virus stability. This further emphasizes the need to use relevant viruses that can be grown under culture conditions that produce droplets similar to those that might be expelled from an infected host. Relying on only Phi6 data could lead to incorrect, and potentially hazardous, conclusions about pathogenic viruses that are more persistent. Strain selection should also be considered when using influenza virus as a surrogate for characterization of emerging virus decay, as previous work has shown that avian influenza viruses undergo more rapid decay than human influenza viruses (23). Decay of enveloped viruses is likely dependent upon many complex changes to the viral glycoprotein after droplet drying or interactions between the glycoprotein and medium composition. Thus, variations in glycoprotein content and density per virus family or strain likely influence the stability within droplets. On the other hand, H1N1pdm09 decayed more similarly to SARS-CoV-2 and could be useful surrogate to extrapolate the latter’s persistence under more physiologically relevant conditions.

One limitation of this study is that we investigated virus persistence in culture medium, DMEM, that may not be representative of real respiratory fluid. We chose to use this medium for the purpose of comparing results with prior studies of SARS-CoV-2 in DMEM (2, 3). Prior studies have shown that virus survival in droplets, including suspended aerosols, is strongly dependent on the chemical composition of the suspending medium (1, 21, 24). In particular, we have previously shown that H1N1pdm09 in aerosols and 1-μL droplets survived better when the suspending medium was supplemented with extracellular material from human bronchial epithelial cells (1). Further studies will be required to characterize whether extracellular material from airway cells alters the biphasic decay patterns (increased decay during the wet phase followed by slower decay during the dry phase) observed in this study.

Extrapolating our results to smaller droplet sizes and combining them with the findings of other studies may provide mechanistic insight into the dynamics of virus inactivation in droplets and aerosols. The biphasic virus decay that is readily observed in droplets likely occurs in aerosols, too (2, 9, 25, 26). While a droplet/aerosol is wet and evaporation is still occurring, the virus is subject to a higher decay rate than after the droplet/aerosol reaches a solid or semisolid state at quasi-equilibrium, as we observed for all droplet sizes tested (5). At the point of efflorescence (the crystallization of salts as water evaporates), if it occurs, there appears to be a step change loss in infectivity. With aerosols, the first phase occurs quickly, within seconds, and further observations of decay are dominated by the quasi-equilibrium phase. Thus, the first phase of decay is important for transmission at close range, when exposure occurs within seconds, while both phases are important for transmission at farther range. While the residence time of aerosols in indoor air will typically not exceed a few hours before they are removed by ventilation, droplets that are deposited on surfaces could remain there for much longer. So, virus on surfaces could persist for longer time scales than virus within aerosols.

Although virus stability in droplets and aerosols appears to be a complex function of droplet size, composition, humidity, and other variables, mechanistically their role is to modulate the microenvironment surrounding a virion (5, 26, 27). Ultimately, molecular-scale interactions are what lead to virus inactivation. We combined results for all droplet sizes and all RHs and plotted virus decay as a function of extent of evaporation of a droplet (Fig. S3), a proxy for its instantaneous physical and chemical characteristics. There appeared to be less separation in results under different experimental conditions than in plots considering initial droplet size (Fig. 2) and RH (Fig. 4). This observation supports our hypothesis that the critical factor controlling virus decay is a virion’s microenvironment and that initial droplet size and RH are indicators of this factor. The exact mechanisms of virus inactivation—the biochemical changes that occur—remain unknown. Determination of viral gene copies in a subset of samples at 1 h and 24 h revealed no appreciable genome loss (Table S7), indicating that genome instability does not contribute to this inactivation. Therefore, virus inactivation mechanisms are ripe for further investigation.

10.1128/mbio.03452-22.3 FIG S3 Evaporation is major determinant of virus decay regardless of initial droplet volume. Log_10_ virus decay was plotted against percent original mass for Phi6, H1N1pdm09, and SARS-CoV-2 in 1 × 50 μL, 5 × 5 μL, and 10 × 1 μL at 40% RH, 65% RH, and 85% RH. Percent original mass was determined by determining droplet mass at 0, 20, and 40 minutes, then 1, 4, 8, and 24 h. RH listed in the legend show the targeted RH. Actual RH data are available in Fig. S2, except for data previously published by van Doremalen et al. (N Engl J Med 382:1564–1567, 2020, https://doi.org/10.1056/NEJMc2004973), for which actual RH values are unavailable. Download FIG S3, PDF file, 0.1 MB. Copyright © 2023 French et al. 2023 French et al. https://creativecommons.org/licenses/by/4.0/ This content is distributed under the terms of the Creative Commons Attribution 4.0 International license.

10.1128/mbio.03452-22.10 TABLE S7 Genome copies for limited SARS-CoV-2 and H1N1pdm09 experiments demonstrate consistent recovery and genome stability. Download Table S7, PDF file, 0.1 MB. Copyright © 2023 French et al. 2023 French et al. https://creativecommons.org/licenses/by/4.0/ This content is distributed under the terms of the Creative Commons Attribution 4.0 International license.

Due to our findings on the sensitivity of virus persistence to both droplet volume and composition, we urge a shift toward the use of more realistic conditions in future studies. They should employ droplets as close in volume as possible to those released from the respiratory tract (submicrometer up to several hundred micrometers in diameter) and whose chemical composition closely mimics that of real respiratory fluid. These findings are critical for pandemic risk assessment of emerging pathogens and useful to improve public policy on optimal transmission mitigation strategies.

## MATERIALS AND METHODS

### Cells and viruses.

MDCK cells (obtained from the ATCC) were grown at 37°C in 5% CO_2_ in minimum essential medium containing 10% fetal bovine serum (FBS), penicillin-streptomycin, and l-glutamine. Influenza A virus A/CA/07/2009 was derived from reverse genetics and grown in MDCK cells for 48 h in DMEM (D6546-500ML; Sigma) containing 2% FBS, antibiotic-antimycotic, and l-glutamine. Stocks were diluted to 10^6^ TCID_50_/mL using DMEM containing 2% FBS, penicillin-streptomycin, and l-glutamine for use in experiments. Virus titers were measured using the 50% tissue culture infectious dose assay on MDCK cells and calculated using the Spearman-Karber method (28).

Phi6 was propagated in Luria-Bertani medium from stock suspensions according to established methods (29). The virus was then ultracentrifuged and resuspended in the same Dulbecco’s modified Eagle medium as previously described using an Optima XPN-100 ultracentrifuge. While ultracentrifugation of virus can cause aggregation, Phi6 cannot be grown in DMEM; ultracentrifugation had to be done to maintain a constant droplet composition. Stocks were diluted to 10^6^ PFU/mL for use in experiments. Virus titers were quantified by plaque assay (22).

SARS-CoV-2 strain USA-WA1/2020 (NR-52281; BEI Resources, Manassas, VA) was passaged in Vero cells once before receipt and subsequently in Vero cells once upon receipt. Vero cells were grown in DMEM (10-017-CV; Corning) supplemented with 5% FBS (97068-086; VWR), 100 units/mL of penicillin, and 100 mg/mL of streptomycin (15140122; Gibco) and maintained at 37°C and 5% CO_2_. Virus titers were quantified by plaque assay as described previously (30). Stocks were diluted to 10^6^ PFU/mL in DMEM (D6546-500ML; Sigma) containing 2% FBS, antibiotic-antimycotic, and l-glutamine for use in experiments. All SARS-CoV-2-related work was conducted in biosafety level 3 (BSL3) laboratories.

Given that TCID_50_ and plaque assay have a linear relationship, virus decay should be comparable between virus quantification methods (30).

### Evaporation experiment.

We measured the evaporation kinetics of DMEM droplets containing Phi6 under the same humidity conditions as in the virus stability experiments. We tested each droplet volume independently by measuring the mass of the droplets every 10 min for up to 24 h using a microbalance (Sartorius; MSE3.6P-000-DM; readability, 0.0010 mg) placed in the environmental chamber.

### Virus stability studies.

We measured virus stability for Phi6 and H1N1pdm09 in a temperature- and humidity-controlled environmental chamber (Electro-Tech Systems) at room temperature and three (40%, 65%, and 85%) or four (also 60% for H1N1pdm09) RHs. A logger (HOBO UX100-011) placed inside the chamber recorded temperature and relative humidity. The temperature and humidity for all experiments are presented in Fig. S2. Briefly, the temperature ranged between 21 and 25°C in all studies and ±5% of the desired RH. The average measured RH was within ±2% of the targeted RH (Fig. S2). The absolute humidities (AH) corresponding to the four targeted RHs (40%, 60%, 65%, and 85%) were approximately 0.008, 0.011, 0.012, and 0.017 kg/m^3^, respectively (Fig. S2I). Droplets (1 × 50 μL, 5 × 5 μL, or 10 × 1 μL) were pipetted onto 6-well polystyrene tissue culture-coated plates (Thermo Scientific) in technical duplicates. Droplets were resuspended at seven different time points (0 min, 20 min, 40 min, 1 h, 4 h, 8 h, and 24 h), or four time points (1, 4, 8, and 24 h) for the experiment at 60% RH, using 500 μL of DMEM containing 2% FBS, penicillin-streptomycin, and l-glutamine.

We measured the stability of SARS-CoV-2 in an airtight desiccator at room temperature and 55% RH as described previously (19). The measured temperature ranged between 23 and 28°C. In short, we filled one polyethylene petri dish with 10 to 20 mL of saturated magnesium nitrate solution and placed it at the bottom of the desiccator to control the humidity. A battery-powered fan was also placed inside to enhance air mixing and thus accelerate the establishment of equilibrium, which was usually within 5 to 10 min. A logger recorded temperature and relative humidity. After RH equilibrium was reached, 2 × 50-μL, 5 × 5-μL, or 10 × 1-μL droplets were deposited and suspended as described previously. Plaque assay on Vero cells was used to measure virus titers.

All H1N1pdm09 and Phi6 collections were performed in technical duplicates and independent triplicates. Due to biosafety constraints and the additional resources required, the SARS-CoV-2 work was performed in technical duplicates with two independent replicates. Data from all replicates are presented for each virus.

### Calculations and modeling.

We measured the initial mass [*m*(0)] immediately after the droplets were deposited onto the polystyrene surface. We calculated the percent original mass of the droplet over time using the following equation:

$$\text{\%}\;\text{Original}\;\text{mass}=100\;-\;\frac{\left[m\left(0\right)\;-\;m\left(t\right)\right]}{m\left(0\right)}\times 100$$

We defined the time to quasi-equilibrium state, also referred to as the drying time, as the time to reach a value that did not increase or decrease by more than 2%.

Virus decay was calculated as follows:

$${\text{log}}_{\text{1}0}\;\text{decay}=\log\frac{N(0)}{N(t)}$$

where *N*(0) is titer at time zero and *N*(*t*) is titer at time *t*. Because virus decay was measured by comparing to titer at time zero, we have already accounted for potential loss due to resuspension.

We modeled the decay of viruses using first-order exponential decay curves separately for the wet phase and the dry phase. The first order decay equation is as follows:

$$N\left(t\right)=N\left(0\right)e^{-\mathrm{kt}}$$

We ran a linear regression in R through the log-transformed equation to find the decay rate. For wet decay, we adjusted for the increasing concentrations of solutes over time, as shown in the following equation (2):

$$\log_{10}\left[N\left(t\right)\right]=\log_{10}\left[N(0)\right]\;+\;\frac{k_{0}}{B}\log_{10}\left(1\;-\;\mathrm{Bt}\right)$$

where *N*(0) is the initial virus titer, *N*(*t*) is the titer at time *t*, *k*_0_ is the initial first-order rate constant during the evaporation phase, and *B* equals the slope of the wet change in mass divided by the initial water mass.

### qRT-PCR.

We performed reverse transcription-quantitative PCR (qRT-PCR) on one independent replicate each of H1N1pdm09 and SARS-CoV-2 to address genome instability as a possible mechanism of inactivation. For SARS-CoV-2 the Qiagen QIAamp Viral RNA minikit was used to isolate RNA according to the manufacturer’s protocol. The 2019-nCoV RUO primer/probe kit targeting the N1 gene (IDT) was used in combination with the iTaq universal probe one-step kit. Synthetic SARS-CoV-2 RNA (BEI Resources) was used as a standard. For H1N1pdm09, the Qiagen QIAamp viral RNA minikit was used to isolate RNA according to the manufacturer’s protocol. The influenza virus M gene was targeted using the iTaq universal probe one-step kit with primers (forward, 5′-AGATGAGTCTTCTAACCGAGGTCG-3′, and reverse, 5′-GCAAAGACACTTTCCAGTCTCTG-3′) and probe (5′-6-carboxyfluorescein [FAM]-TCAGGCCCCCTCAAAGCCGA-3BHQ1-3′). *In vitro*-transcribed RNA served as standards to determine genome copies. Each qPCR plate contained duplicates of standards, samples, and no-template controls.

### Multivariate analysis.

We conducted a multivariate analysis of variance (MANOVA) to determine if there was an interaction between initial droplet volume and RH that would account for differences in the wet phase decay of the viruses. There was no statistically significant amount of variance (*P* = 0.339) for Phi6 wet-phase decay, but there was statistically significant variance for H1N1pdm09 wet-phase decay (*P* = 0.037), indicating that there is an interaction between initial droplet volume and RH that affects wet-phase decay.

### Data availability.

All raw data associated with this study are available on Figshare at https://doi.org/10.6084/m9.figshare.c.6458767. In addition, movies corresponding to the droplet drying over time at 3 different relative humidities can be found within the collection and at these individual links: https://doi.org/10.6084/m9.figshare.21711119, https://doi.org/10.6084/m9.figshare.21711122, and https://doi.org/10.6084/m9.figshare.21711116.
