# Supplementary material for: Environmental Stability of Enveloped Viruses Is Impacted by Initial Volume and Evaporation Kinetics of Droplets
Source: mBio. 2023 Apr 10;14(2):e03452-22. doi: 10.1128/mbio.03452-22 (PMC10128059; doi:10.1128/mbio.03452-22)
Supplement: FIG S1 [file mbio.03452-22-s0001.pdf]

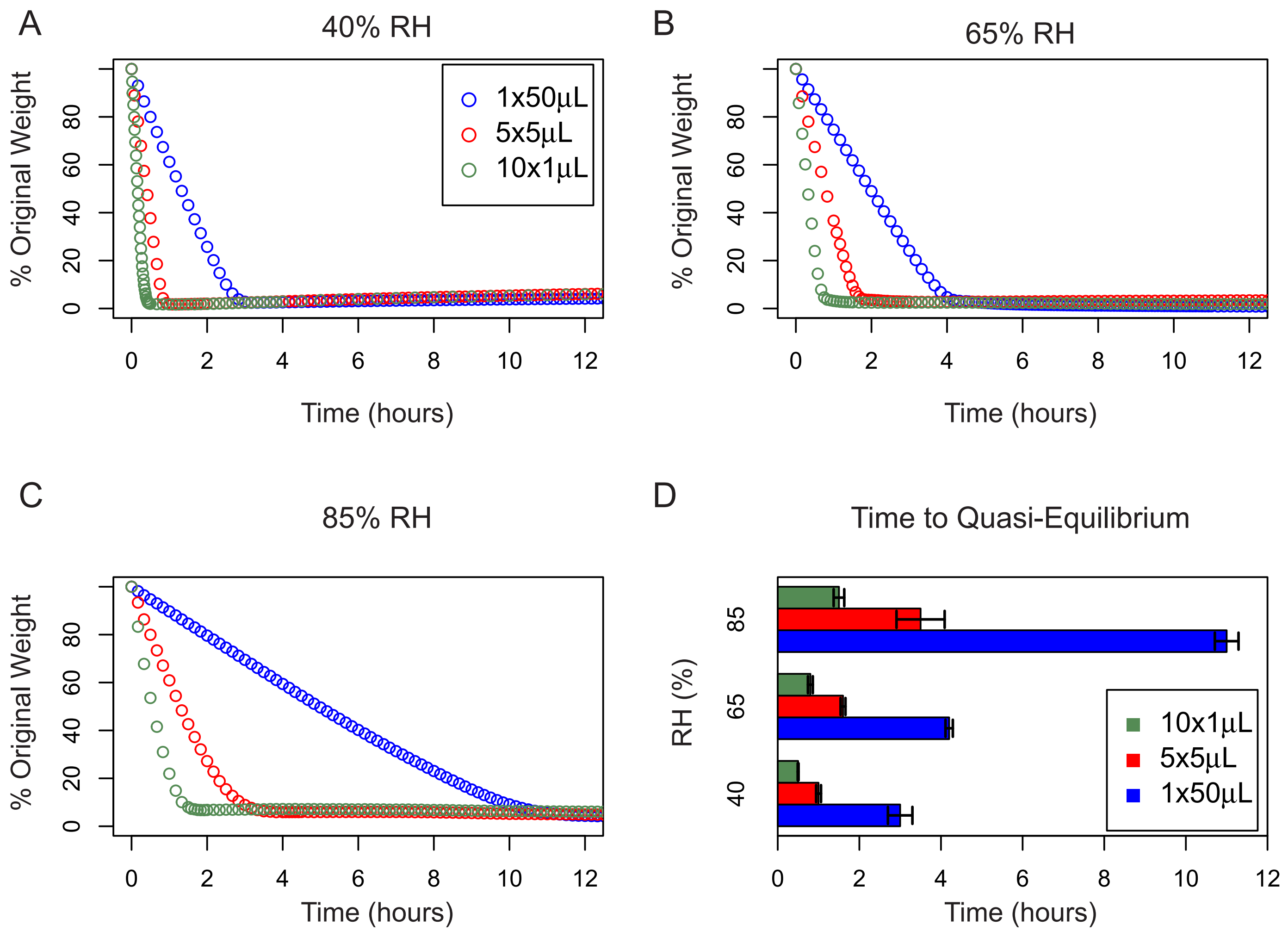

Supplemental Figure 1. Initial droplet volume impacts drying kinetics. A-C. Mass normalized to starting mass for all droplet volumes at **(A)** 40%, **(B)** 65%, **(C)** or 85% RH over time. **D.** Summary data showing the time (mean and standard deviation, n = 2) for droplets to reach quasi-equilibrium at each RH. Droplet mass was measured on a micro-balance in an environmental chamber and recorded every minute.
