# Supplementary material for: Environmental Stability of Enveloped Viruses Is Impacted by Initial Volume and Evaporation Kinetics of Droplets
Source: mBio. 2023 Apr 10;14(2):e03452-22. doi: 10.1128/mbio.03452-22 (PMC10128059; doi:10.1128/mbio.03452-22)
Supplement: TABLE S1 [file mbio.03452-22-s0004.pdf]

Supplementary Material for "Environmental Stability of Enveloped Viruses is Impacted by the Initial Volume and Evaporation Kinetics of Droplets"

Andrea J. French, BS[1*], Alexandra K. Longest, BS[2*], Jin Pan, PhD[2], Peter J. Vikesland, PhD[2], Nisha K. Duggal, PhD[3], Seema S. Lakdawala, PhD[1,4+], Linsey C. Marr, PhD[2+]

Supplemental Table 1. The evaporation rates for 1x50 µL, 5x5 µL, or 10x1 µL droplets at 40%, 65%, or 85% RH were determined by fitting a line to the mass over time.

**RH (%)**	**Initial Volume (µL)**	**Slope ± Std. Error (mg/hour)**
40	50	17.9482 ± 0.164
	5	29.9237 ± 0.376
	1	30.1360 ± 0.438
65	50	12.3290 ± 0.102
	5	15.0284 ± 0.301
	1	18.6950 ± 0.601
85	50	5.0560 ± 0.009
	5	8.8678 ± 0.105
	1	10.7260 ± 0.417
