# Supplementary material for: Environmental Stability of Enveloped Viruses Is Impacted by Initial Volume and Evaporation Kinetics of Droplets
Source: mBio. 2023 Apr 10;14(2):e03452-22. doi: 10.1128/mbio.03452-22 (PMC10128059; doi:10.1128/mbio.03452-22)
Supplement: TABLE S2 [file mbio.03452-22-s0005.pdf]

Supplemental Table 2. 1x50 µL, 5x5 µL, or 10x1 µL droplets were weighed over time. The time of quasi-equilibrium was determined and the mass at quasi-equilibrium was measured.

RH (%)	Initial Volume (µL)	Mean Time at Quasi-Equilibrium ± Std. Error (hours)	Mean Mass at Quasi-Equilibrium. ± Std. Error	
			mg	% Original
40	50	3.0 ± 0.30	1.392 ± 0.38	2.805 ± 0.56
	5	1.0 ± 0.06	0.425 ± 0.15	1.731± 0.74
	1	0.5 ± 0.01	0.202 ± 0.07	1.994 ± 0.82
65	50	4.2 ± 0.09	1.787 ± 0.05	3.559 ± 0.33
	5	1.6 ± 0.06	1.037 ± 0.05	4.044 ± 0.32
	1	0.8 ± 0.06	0.487 ± 0.06	3.699 ± 0.32
85	50	11 ± 0.30	2.813 ± 0.05	5.565 ± 0.09
	5	3.5 ± 0.59	1.492 ± 0.32	6.273 ± 1.29
	1	1.5 ± 0.13	0.978 ± 0.03	7.983 ± 0.415
