# Supplementary material for: Environmental Stability of Enveloped Viruses Is Impacted by Initial Volume and Evaporation Kinetics of Droplets
Source: mBio. 2023 Apr 10;14(2):e03452-22. doi: 10.1128/mbio.03452-22 (PMC10128059; doi:10.1128/mbio.03452-22)
Supplement: FIG S2 [file mbio.03452-22-s0002.pdf]

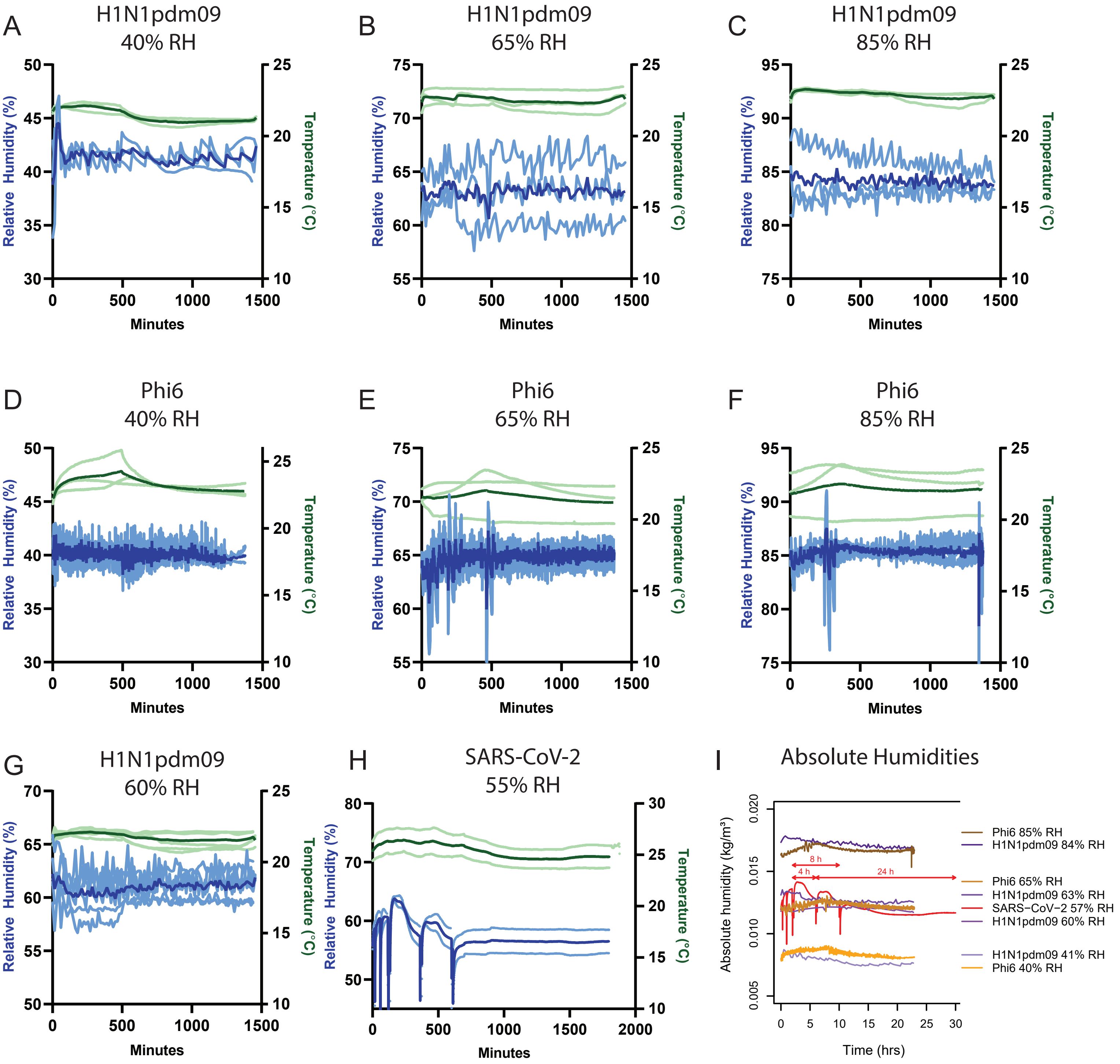

Supplemental Figure 2. Environmental conditions of H1N1pdm09 droplets and Phi6 droplets were within 5% of targeted RH and maintained temperatures between 20 and 28ºC. The RH and temperature of the environmental chamber were recorded every 15 minutes during H1N1pdm09 stability experiments at (**A**) 40%, (**B**) 65%, (**C**) and 85% RH. The RH and temperature of the environmental chamber were recorded every minute during Phi6 stability experiments at (**D**) 40%, (**E**) 65%, (**F**) and 85% RH. The RH and temperature data for comparing decay of (**G**) H1N1pdm09 at 60% with (**H**) SARS-CoV-2 at 55% were recorded every 15 minutes or 1 minute, respectively. Light green shows the temperature at each replicate, and dark green indicates the average temperature for the 3 independent replicates. Light blue shows the RH for each replicate, and dark blue indicates the average RH for the 3 independent replicates. **I**. Absolute humidities were calculated for all experiments. The legends show the corresponding average RH for each condition instead of targeted RH. Conditions for data published in van Dorelamen et al[3] are unknown
