# Supplementary material for: Environmental Stability of Enveloped Viruses Is Impacted by Initial Volume and Evaporation Kinetics of Droplets
Source: mBio. 2023 Apr 10;14(2):e03452-22. doi: 10.1128/mbio.03452-22 (PMC10128059; doi:10.1128/mbio.03452-22)
Supplement: TABLE S4 [file mbio.03452-22-s0007.pdf]

Supplemental Table 4. Log_{10} decay for each virus was compared to 0 decay at each time point in 1x50 µL droplets.

**Virus**	**RH (%)**	**Time**	**p-value**
Phi6	40	0	NA
		0.33	0.15
		0.67	0.42
		1	0.014*
		4	<0.01*
		8	<0.001*
		24	<0.001*
	65	0	NA
		0.33	0.78
		0.67	0.66
		1	0.037*
		4	0.018*
		8	<0.01*
		24	<0.01*
	85	0	NA
		0.33	0.040*
		0.67	0.56
		1	0.19
		4	0.057
		8	0.025*
		24	<0.01*
H1N1pdm09	40	0	NA
		0.33	0.42
		0.67	0.83
		1	0.58
		4	<0.01*
		8	<0.01*
		24	<0.01*
	65	0	NA
		0.33	0.42
		0.67	0.84
		1	0.73
		4	0.30
		8	<0.01*
		24	<0.001*
	85	0	NA
		0.33	0.67
		0.67	1
		1	0.13
		4	0.057
		8	0.069
		24	<0.01*

A t-test was used to determine statistical significance.
