# Supplementary material for: Environmental Stability of Enveloped Viruses Is Impacted by Initial Volume and Evaporation Kinetics of Droplets
Source: mBio. 2023 Apr 10;14(2):e03452-22. doi: 10.1128/mbio.03452-22 (PMC10128059; doi:10.1128/mbio.03452-22)
Supplement: TABLE S3 [file mbio.03452-22-s0006.pdf]

**Supplemental Table 3**. $Log_{10}$ decay for each virus was compared between RH for each droplet volume and p-values were determined.

| Virus | Initial Volume (μL) | Time (hr) | RH 1 (%) | RH 2 (%) | p-value |
|---|---|---|---|---|---|
| Phi6 | 50 | 0.33 | 40 | 65 | 0.074 |
| | | 0.33 | 40 | 85 | 0.42 |
| | | 0.33 | 65 | 85 | 0.40 |
| | | 0.67 | 40 | 65 | 0.79 |
| | | 0.67 | 40 | 85 | 0.99 |
| | | 0.67 | 65 | 85 | 0.84 |
| | | 1 | 40 | 65 | 0.033* |
| | | 1 | 40 | 85 | 0.014* |
| | | 1 | 65 | 85 | 0.75 |
| | | 4 | 40 | 65 | <0.001* |
| | | 4 | 40 | 85 | <0.001* |
| | | 4 | 65 | 85 | 0.014* |
| | | 8 | 40 | 65 | 0.59 |
| | | 8 | 40 | 85 | <0.001* |
| | | 8 | 65 | 85 | <0.001* |
| | | 24 | 40 | 65 | 0.65 |
| | | 24 | 40 | 85 | 0.21 |
| | | 24 | 65 | 85 | 0.59 |
| Phi6 | 5 | 0.33 | 40 | 65 | 0.51 |
| | | 0.33 | 40 | 85 | 0.88 |
| | | 0.33 | 65 | 85 | 0.78 |
| | | 0.67 | 40 | 65 | 0.18 |
| | | 0.67 | 40 | 85 | 0.59 |
| | | 0.67 | 65 | 85 | 0.60 |
| | | 1 | 40 | 65 | 0.17 |
| | | 1 | 40 | 85 | 0.037* |
| | | 1 | 65 | 85 | 0.49 |
| | | 4 | 40 | 65 | 0.16 |
| | | 4 | 40 | 85 | 0.84 |
| | | 4 | 65 | 85 | 0.33 |
| | | 8 | 40 | 65 | 0.27 |
| | | 8 | 40 | 85 | 0.78 |
| | | 8 | 65 | 85 | 0.58 |
| | | 24 | 40 | 65 | 0.70 |
| | | 24 | 40 | 85 | 0.23 |
| | | 24 | 65 | 85 | 0.58 |
| Phi6 | 1 | 0.33 | 40 | 65 | 0.66 |
| | | 0.33 | 40 | 85 | 0.94 |
| | | 0.33 | 65 | 85 | 0.47 |
| | | 0.67 | 40 | 65 | 0.015* |
| | | 0.67 | 40 | 85 | 0.002* |
| | | 0.67 | 65 | 85 | 0.13 |
| | | 1 | 40 | 65 | 0.036* |
| | | 1 | 40 | 85 | <0.001* |
| | | 1 | 65 | 85 | 0.020* |
| | | 4 | 40 | 65 | 0.012* |
| | | 4 | 40 | 85 | 1.0 |
| | | 4 | 65 | 85 | 0.012* |
| | | 8 | 40 | 65 | 0.050 |
| | | 8 | 40 | 85 | 0.72 |
| | | 8 | 65 | 85 | 0.14 |
| | | 24 | 40 | 65 | 0.78 |
| | | 24 | 40 | 85 | 0.062 |
| | | 24 | 65 | 85 | 0.15 |
| H1N1pdm09 | 50 | 0.33 | 40 | 65 | 0.44 |
| | | 0.33 | 40 | 85 | 1 |
| | | 0.33 | 65 | 85 | 0.44 |
| | | 0.67 | 40 | 65 | 0.92 |
| | | 0.67 | 40 | 85 | 0.98 |
| | | 0.67 | 65 | 85 | 0.98 |
| | | 1 | 40 | 65 | 0.98 |
| | | 1 | 40 | 85 | 0.94 |
| | | 1 | 65 | 85 | 0.86 |
| | | 4 | 40 | 65 | 0.005* |
| | | 4 | 40 | 85 | 0.004* |
| | | 4 | 65 | 85 | 0.96 |
| | | 8 | 40 | 65 | 0.096 |
| | | 8 | 40 | 85 | <0.001* |

| | | | | | |
|---|---|---|---|---|---|
| | | 8 | 65 | 85 | <0.001* |
| | | 24 | 40 | 65 | 0.012* |
| | | 24 | 40 | 85 | 0.015* |
| | | 24 | 65 | 85 | 0.97 |
| H1N1pdm09 | 5 | 0.33 | 40 | 65 | 0.65 |
| | | 0.33 | 40 | 85 | 0.97 |
| | | 0.33 | 65 | 85 | 0.53 |
| | | 0.67 | 40 | 65 | 0.32 |
| | | 0.67 | 40 | 85 | 0.065 |
| | | 0.67 | 65 | 85 | 0.46 |
| | | 1 | 40 | 65 | 0.96 |
| | | 1 | 40 | 85 | 0.68 |
| | | 1 | 65 | 85 | 0.82 |
| | | 4 | 40 | 65 | 0.61 |
| | | 4 | 40 | 85 | 0.34 |
| | | 4 | 65 | 85 | 0.85 |
| | | 8 | 40 | 65 | 0.85 |
| | | 8 | 40 | 85 | 0.34 |
| | | 8 | 65 | 85 | 0.17 |
| | | 24 | 40 | 65 | 0.47 |
| | | 24 | 40 | 85 | 0.38 |
| | | 24 | 65 | 85 | 0.98 |
| H1N1pdm09 | 1 | 0.33 | 40 | 65 | 1 |
| | | 0.33 | 40 | 85 | 0.99 |
| | | 0.33 | 65 | 85 | 0.99 |
| | | 0.67 | 40 | 65 | 0.97 |
| | | 0.67 | 40 | 85 | 0.60 |
| | | 0.67 | 65 | 85 | 0.48 |
| | | 1 | 40 | 65 | 0.82 |
| | | 1 | 40 | 85 | 0.74 |
| | | 1 | 65 | 85 | 0.41 |
| | | 4 | 40 | 65 | 0.53 |
| | | 4 | 40 | 85 | 0.77 |
| | | 4 | 65 | 85 | 0.90 |
| | | 8 | 40 | 65 | 0.43 |
| | | 8 | 40 | 85 | 0.73 |
| | | 8 | 65 | 85 | 0.85 |
| | | 24 | 40 | 65 | 0.82 |
| | | 24 | 40 | 85 | 0.82 |
| | | 24 | 65 | 85 | 1 |

A one-way ANOVA and Tukey HSD test was used to determine significance.
