# Supplementary material for: Environmental Stability of Enveloped Viruses Is Impacted by Initial Volume and Evaporation Kinetics of Droplets
Source: mBio. 2023 Apr 10;14(2):e03452-22. doi: 10.1128/mbio.03452-22 (PMC10128059; doi:10.1128/mbio.03452-22)
Supplement: TABLE S5 [file mbio.03452-22-s0008.pdf]

Supplemental Table 5. Decay constants for each phase and virus were compared within each RH and droplet volume to characterize how phase and virus impact virus decay.

Initial Volume (μL)	RH (%)	Virus 1	Virus 2	Phase 1	Phase 2	p-value
50	40	Phi6	Phi6	Evaporation	Dry	0.059
5	40	Phi6	Phi6	Evaporation	Dry	0.037*
1	40	Phi6	Phi6	Evaporation	Dry	NA
50	40	H1N1pdm09	H1N1pdm09	Evaporation	Dry	0.37
5	40	H1N1pdm09	H1N1pdm09	Evaporation	Dry	0.045*
1	40	H1N1pdm09	H1N1pdm09	Evaporation	Dry	NA
50	40	Phi6	H1N1pdm09	Evaporation	Evaporation	0.12
50	40	Phi6	H1N1pdm09	Dry	Dry	<0.01*
5	40	Phi6	H1N1pdm09	Evaporation	Evaporation	0.41
5	40	Phi6	H1N1pdm09	Dry	Dry	0.86
1	40	Phi6	H1N1pdm09	Evaporation	Evaporation	NA
1	40	Phi6	H1N1pdm09	Dry	Dry	0.71
50	65	Phi6	Phi6	Evaporation	Dry	NA
5	65	Phi6	Phi6	Evaporation	Dry	0.044*
1	65	Phi6	Phi6	Evaporation	Dry	0.69
50	65	H1N1pdm09	H1N1pdm09	Evaporation	Dry	NA
5	65	H1N1pdm09	H1N1pdm09	Evaporation	Dry	0.98
1	65	H1N1pdm09	H1N1pdm09	Evaporation	Dry	0.15
50	65	Phi6	H1N1pdm09	Evaporation	Evaporation	<0.001*
50	65	Phi6	H1N1pdm09	Dry	Dry	NA
5	65	Phi6	H1N1pdm09	Evaporation	Evaporation	0.39
5	65	Phi6	H1N1pdm09	Dry	Dry	0.036*
1	65	Phi6	H1N1pdm09	Evaporation	Evaporation	0.22
1	65	Phi6	H1N1pdm09	Dry	Dry	0.77
50	85	Phi6	Phi6	Evaporation	Dry	NA
5	85	Phi6	Phi6	Evaporation	Dry	0.24
1	85	Phi6	Phi6	Evaporation	Dry	0.76
50	85	H1N1pdm09	H1N1pdm09	Evaporation	Dry	NA
5	85	H1N1pdm09	H1N1pdm09	Evaporation	Dry	0.30
1	85	H1N1pdm09	H1N1pdm09	Evaporation	Dry	0.23
50	85	Phi6	H1N1pdm09	Evaporation	Evaporation	<0.001*
50	85	Phi6	H1N1pdm09	Dry	Dry	NA
5	85	Phi6	H1N1pdm09	Evaporation	Evaporation	0.43
5	85	Phi6	H1N1pdm09	Dry	Dry	0.14
1	85	Phi6	H1N1pdm09	Evaporation	Evaporation	0.15
1	85	Phi6	H1N1pdm09	Dry	Dry	0.48

A t-test was used to determine statistical significance.
