# Supplementary material for: Environmental Stability of Enveloped Viruses Is Impacted by Initial Volume and Evaporation Kinetics of Droplets
Source: mBio. 2023 Apr 10;14(2):e03452-22. doi: 10.1128/mbio.03452-22 (PMC10128059; doi:10.1128/mbio.03452-22)
Supplement: TABLE S6 [file mbio.03452-22-s0009.pdf]

Supplemental Table 6. Log_{10} decay within 1x50 µL droplets was compared between Phi6, H1N1pdm09, and SARS-CoV-2 at 40%, 65%, and 85% RH over time. Log_{10} decay within 50 µL, 5 µL, or 1 µL droplets was compared between H1N1pdm09 and SARS-CoV-2. SARS-CoV-2 data that was previously published[3] was also compared to original SARS-CoV-2 data.

RH (%)	Time (hours)	Droplet Volume (µL)	Virus 1	Virus 2	p-value
40	0.33	50	Phi6	H1N1pdm09	0.17
	0.67	50	Phi6	H1N1pdm09	0.52
	1	50	Phi6	H1N1pdm09	0.42
		50	Phi6	SARS-CoV-2 (van Doremalen et al[3])	0.67
		50	H1N1pdm09	SARS-CoV-2 (van Doremalen et al[3])	0.15
	4	50	Phi6	H1N1pdm09	<0.001*
		50	Phi6	SARS-CoV-2 (van Doremalen et al[3])	<0.001*
		50	H1N1pdm09	SARS-CoV-2 (van Doremalen et al[3])	0.021*
	8	50	Phi6	H1N1pdm09	<0.001*
		50	Phi6	SARS-CoV-2 (van Doremalen et al[3])	<0.001*
		50	H1N1pdm09	SARS-CoV-2 (van Doremalen et al[3])	<0.01*
65	0.33	50	Phi6	H1N1pdm09	0.42
	0.67	50	Phi6	H1N1pdm09	0.88
	1	50	Phi6	H1N1pdm09	0.69
		50	Phi6	SARS-CoV-2 (van Doremalen et al[3])	0.087
		50	H1N1pdm09	SARS-CoV-2 (van Doremalen et al[3])	0.25
	4	50	Phi6	H1N1pdm09	0.024*
		50	Phi6	SARS-CoV-2 (van Doremalen et al[3])	<0.01*
		50	H1N1pdm09	SARS-CoV-2 (van Doremalen et al[3])	0.24
	8	50	Phi6	H1N1pdm09	<0.001*
		50	Phi6	SARS-CoV-2 (van Doremalen et al[3])	<0.001*
		50	H1N1pdm09	SARS-CoV-2 (van Doremalen et al[3])	0.086
85	0.33	50	Phi6	H1N1pdm09	0.55
	0.67	50	Phi6	H1N1pdm09	0.59
	1	50	Phi6	H1N1pdm09	1.0
		50	Phi6	SARS-CoV-2 (van Doremalen et al[3])	0.68
		50	H1N1pdm09	SARS-CoV-2 (van Doremalen et al[3])	0.71
	4	50	Phi6	H1N1pdm09	0.80
		50	Phi6	SARS-CoV-2 (van Doremalen et al[3])	0.92
		50	H1N1pdm09	SARS-CoV-2 (van Doremalen et al[3])	0.58
	8	50	Phi6	H1N1pdm09	0.51
		50	Phi6	SARS-CoV-2 (van Doremalen et al[3])	0.86
		50	H1N1pdm09	SARS-CoV-2 (van Doremalen et al[3])	0.81
55-60	1	50	H1N1pdm09	SARS-CoV-2 (VT)	0.847
	4	50	H1N1pdm09	SARS-CoV-2 (VT)	0.095
	8	50	H1N1pdm09	SARS-CoV-2 (VT)	0.571
	1	5	H1N1pdm09	SARS-CoV-2 (VT)	0.729
	4	5	H1N1pdm09	SARS-CoV-2 (VT)	0.409
	8	5	H1N1pdm09	SARS-CoV-2 (VT)	0.5

	1	1	H1N1pdm09	SARS-CoV-2 (VT)	0.117
	4	1	H1N1pdm09	SARS-CoV-2 (VT)	0.0712
	8	1	H1N1pdm09	SARS-CoV-2 (VT)	0.822
55-65	1	50	SARS-CoV-2 (van Doremalen et al[3])	SARS-CoV-2 (VT)	0.393
	4	50	SARS-CoV-2 (van Doremalen et al[3])	SARS-CoV-2 (VT)	0.426
	8	50	SARS-CoV-2 (van Doremalen et al[3])	SARS-CoV-2 (VT)	0.0169*
	24	50	SARS-CoV-2 (van Doremalen et al[3])	SARS-CoV-2 (VT)	<0.001*

A one-way ANOVA and Tukey TSD test were used to determine statistical significance. SARS-CoV-2 (van Doremalen et al[3]) data was originally published in van Doremalen et al.[3] SARS-CoV-2 (VT) data is original data collected by JP and NKD.
