# Supplementary material for: Environmental Stability of Enveloped Viruses Is Impacted by Initial Volume and Evaporation Kinetics of Droplets
Source: mBio. 2023 Apr 10;14(2):e03452-22. doi: 10.1128/mbio.03452-22 (PMC10128059; doi:10.1128/mbio.03452-22)
Supplement: FIG S3 [file mbio.03452-22-s0003.pdf]

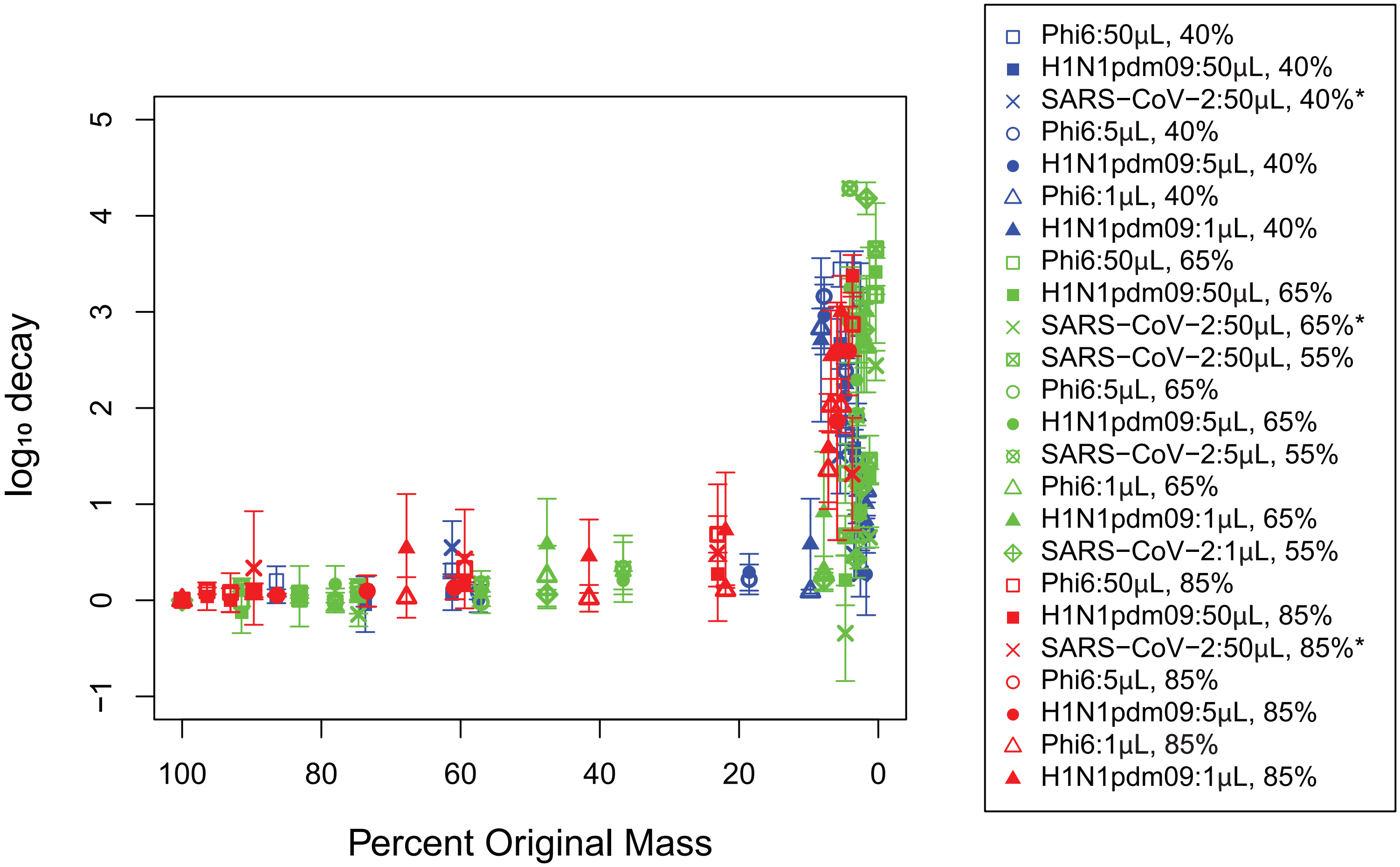

Supplemental Figure 3. Evaporation is major determinant of virus decay regardless of initial droplet volume. Log_{10} virus decay was plotted against percent original mass for Phi6, H1N1pdm09, and SARS-CoV-2 in 50 µL, 5 µL, and 1 µL droplets at 40% RH, 55%, 65% RH, and 85% RH. Percent original mass was determined by determining droplet mass at 0, 20, and 40 minutes, then 1, 4, 8, and 24 hours. RH listed in the legend show the targeted RH. Actual RH are available in Supplemental Figure 2, except for data previously published in van Doremalen et al[3], which is unavailable.
