# Supplementary material for: Environmental Stability of Enveloped Viruses Is Impacted by Initial Volume and Evaporation Kinetics of Droplets
Source: mBio. 2023 Apr 10;14(2):e03452-22. doi: 10.1128/mbio.03452-22 (PMC10128059; doi:10.1128/mbio.03452-22)
Supplement: TABLE S7 [file mbio.03452-22-s0010.pdf]

Supplemental Table 7. Genome copies for limited SARS-CoV-2 and H1N1pdm09 experiments demonstrate consistent recovery and genome stability.

Virus	RH (%)	Time (hours)	Droplet volume (μL)	Log_{10} Genome Copies (per mL)+	Log_{10} Infectious Units (per mL)*	Log_{10} Decay of Infectious Units*
H1N1pdm09	40	1	50	7.61	4.64	0.06
		24	50	7.89	2.03	2.67
		1	5	7.23	4.22	0.27
		24	5	7.51	1.53	2.96
		1	1	6.78	3.20	1.00
		24	1	7.00	1.49	2.71
	60	1	50	7.70	4.70	0.38
		24	50	7.80	2.04	3.03
		1	5	7.41	4.03	0.61
		24	5	7.43	0.82	3.88
		1	1	6.82	3.20	0.88
		24	1	6.86	0.46	3.58
	65	1	50	7.71	4.66	0.04
		24	50	7.61	1.28	3.42
		1	5	7.36	4.28	0.21
		24	5	7.39	1.24	3.25
		1	1	6.92	2.97	1.23
		24	1	6.94	1.20&	3.00
	85	1	50	7.72	4.60	0.10
		24	50	7.46	1.33	3.38
		1	5	7.44	4.43	0.13
		24	5	7.17	1.20&	2.59
		1	1	6.77	3.47	0.73
		24	1	6.93	1.20&	3.00
SARS-CoV-2	55	1	50	8.12	5.40	0.07
		24	50	7.90	1.83	3.66
		1	5	7.42	4.66	0.33
		24	5	7.11	0.70&	4.28
		1	1	6.87	4.22	0.43
		24	1	6.92	0.70&	3.88

H1N1pdm09 infectious units are in $TCID_{50}$/mL while SARS-CoV-2 infectious units are in PFU/mL.
+A cross symbol indicates samples isolated from one replicate.
*The asterisk symbol indicates average data as shown in the main text.
&The ampersand symbol indicates values at the limit of detection
